# Associations of Geographic Access to Special Supplemental Nutrition Program for Women, Infants, and Children–Approved Vendors with Special Supplemental Nutrition Program for Women, Infants, and Children Benefit Redemption in California

**DOI:** 10.1016/j.cdnut.2026.109558

**Published:** 2026-10-23

**Authors:** Christopher E Anderson, Alana M Chaney, Shannon E Whaley, Hussain Chharawalla, Lauren E Au

**Affiliations:** 1 Department of Nutrition and Public Health Sciences, University of Tennessee, Knoxville, TN, United States; 2 Division of Research and Evaluation, Public Health Foundation Enterprises WIC, A Program of Heluna Health, City of Industry, CA, United States; 3 Department of Nutrition, University of California Davis, Davis, CA, United States

**Keywords:** food environment, WIC, benefit redemption, nutrition assistance, geographic access, vendors

## Abstract

**Background:**

Redemption of Special Supplemental Nutrition Program for Women, Infants, and Children (WIC) food benefits is highly variable and often incomplete. How geographic access to WIC-approved vendors contributes to WIC benefit utilization is unknown.

**Objectives:**

This study aimed to assess relationships between geographic access to WIC-approved vendors and WIC benefit utilization among respondents to the 2023 California Statewide WIC Survey (*n*, unweighted = 2201).

**Methods:**

Geographic access to WIC-approved vendors from participants’ residences was assessed as the density (vendors/mile^2^) in buffers of variable radii (1–5 miles) and the proximity (miles) of the nearest 5 vendors. Monthly WIC benefit redemption percentage was calculated and averaged across the 12 mo before the survey for specific benefit categories (e.g., eggs) and across all categories for overall redemption. Associations of density of WIC-approved vendors in 1- to 5-mile buffer radii and proximity of the nearest 5 vendors with benefit redemption (overall and category-specific) were assessed in multivariable generalized estimating equation linear regression models.

**Results:**

Higher vendor density in a 2-mile radius buffer around the residence was associated with higher redemption percentages for overall food benefits {estimate 5.5% [95% confidence interval (CI): 0.6%, 10.4%]}, legumes [7.2% (95% CI: 1.4%, 12.9%)], whole grains [7.6% (95% CI: 1.7%, 13.5%)], and yogurt [6.2% (95% CI: 0.7%, 11.7%)]. Higher vendor density in 1- and 4-mile radius buffers was also associated with higher yogurt redemption. No consistent associations were found for the proximity of the nearest 5 vendors.

**Conclusions:**

Understanding how WIC vendor access contributes to benefit redemption may help WIC programs support more complete benefit redemption to better support food security and healthier diets among participants.

## Introduction

Childhood obesity has become a serious public health challenge affecting 13% of United States children aged 2 to 5 [1], increasing their long-term risk of type 2 diabetes, cardiovascular disease, and certain cancers [2], [3]. As childhood obesity disproportionately impacts children living in low-income households, ∼1 in 4 of whom have obesity [4], identifying strategies to improve diet quality among this population is critical. The Special Supplemental Nutrition Program for Women, Infants, and Children (WIC) provides access to healthy foods through monthly food packages to >6 million low-income participants, in addition to nutrition education and referrals to other health services [5]. The nutritious WIC food package includes items such as eggs, whole grains/bread, milk, legumes, breakfast cereal, 100% juice, and a cash benefit for purchasing fruits and vegetables [6]. Numerous benefits of WIC participation on diet quality have been shown, including lower consumption of sugar-sweetened beverages [7] and lower obesity risk [8]. Although there are many health benefits from being in the WIC program, prior evidence suggests that not all of the WIC food package is redeemed [9], [10], [11]. Low redemption of cereal, whole grains, yogurt, whole milk, and 100% juice is associated with lower intake of these foods [12], and lower redemption of WIC foods (specific foods and overall) is associated with greater risk of discontinued WIC participation [10], [13]. Understanding the factors contributing to participant benefit redemption can inform interventions that can maximize the WIC program’s impact on nutritional outcomes in young children.

Because WIC benefits must be redeemed in person at a WIC-approved vendor, geographic access to vendors may contribute to benefit redemption patterns, and in 2019, 40% of all individuals lived >1 mile from the nearest food store [14]. It is recognized that the food environment and access to nutritious foods may be key contributors to diet quality [15], though associations between supermarket access with diet [16], [17] and weight outcomes [18], [19], [20] have been mixed and modestly sized. Low-income households with limited access to supermarkets and grocery stores tend to purchase fewer perishable foods, such as fresh fruits and vegetables, likely due to the increased travel burden, although overall food purchasing is only modestly influenced by supermarket access when food price is taken into account [21]. For instance, while changes to the WIC food package in 2009 were associated with significantly reduced obesity risk among participating children, the neighborhood food environment modified this relationship, suggesting that preschool-aged girls living in areas with a greater density of unhealthy food outlets did not equally benefit from this improved food package [22]. Further, WIC participants have reported that transportation challenges, limited available WIC-approved vendors, and limited stock of WIC-approved items at local stores impact their benefit redemption and program participation [11], [23], [24]. Determining effective geographic access measures and examining their direct link to benefit redemption can support future efforts to expand access for WIC participants.

Limited research exists in quantifying this association between participants’ access to WIC vendors and benefit redemption. In a Los Angeles County cohort, WIC-participating preschool-aged children living in census tracts with ∼0.7 healthy food outlets per mile^2^ had lower measures of adiposity than those living in areas with higher or lower healthy food outlet densities [20]. This work suggests a moderating role of local food access among WIC-participating children, yet was not able to capture household redemption of WIC food benefits or characterize outlets as WIC vendors. Therefore, the primary objective of this study was to examine the association between WIC vendor access and benefit redemption among WIC-participating families with children across California. A secondary aim was to determine the most effective measure of vendor access, such as proximity or geographic density. It was hypothesized that households with greater access to WIC vendors would have higher benefit redemption.

## Methods

### Sample and setting

Data used in this study were collected as part of the bimodal (telephone and online) 2023 California Statewide WIC Survey. The sampling frame was 10,000 WIC-participating households selected at random from study-eligible households in California in January 2023. To be survey-eligible, a household was required to have a nonfoster child WIC participant aged 1 to 4 y and to have an English or Spanish language preference. Recruitment letters were mailed to eligible households until the target sample size of 3000 completed surveys was reached (2129 telephone, 871 internet). Letters included a quick response code and access code to allow the household to complete the survey online if they preferred that before they were called and offered an opportunity to complete a telephone survey. An incentive of $10 was provided for completing the survey. The survey response rate was 46%. It was calculated using the number of completed surveys (numerator) divided by the sum of the number of completed surveys and the number of nonresponses [refusals, call blocking, and language barriers plus the proportion of survey eligibility among those who agreed to complete the survey (0.98)] multiplied by the number of nonresponses due to call blocking and unavailability for the full study period. Poststratification weights were calculated to ensure that responses were representative of the racial, ethnic, language, child age, California census region, and household WIC benefit redemption of the study-eligible population [12], [25]. More details on the survey recruitment and statistical weights have been reported previously [12], [26]. Children with missing data on study variables were excluded from the present analysis, leaving a final analytic sample of 2201 children (*n*, weighted = 2173). The response rate was 33% when calculated for the final analytic sample, which is comparable to the response rate for the 2019 California Statewide WIC Survey [27]. The California Health and Human Services Agency Committee for the Protection of Human Subjects, acting as an Institutional Review Board, approved this study (protocol 23-025 on 3 March, 2023), and informed consent was obtained from all respondents (verbal for telephone surveys, written for internet surveys).

### WIC administrative data

WIC administrative data, collected during routine provision of WIC services, include data on participant and household sociodemographic characteristics and on WIC food benefit issuance and redemption. Household WIC benefit redemption was the primary outcome for this study. WIC food benefits are issued and redeemed on a rolling monthly basis (unredeemed benefits expire at the end of the 1-mo period in which the benefits must be redeemed) in categories (10 separate categories for the present study: cheese/tofu, eggs, breakfast cereal, legumes, bread/whole grains, fruits and vegetables, yogurt, whole milk, reduced-fat milk, and 100% juice). Redemption percentages were calculated for each food benefit category each month for every family by dividing the amount redeemed by the amount issued and multiplying by 100. Overall benefit redemption percentage was calculated each month by averaging across all food benefit categories that a family was issued in a given month. Overall and food benefit category-specific redemption percentages were then averaged for all months with nonmissing issuance across the 12 mo before completing the survey.

### Geographic access to WIC-approved vendors

The primary exposure of interest for this analysis was the geographic access to WIC-approved vendors around the residence of survey respondents. The geographic location of all vendors approved by the California WIC program was determined using geographic information systems. The precise location of the residence of each respondent to the survey was determined using participant addresses, and the distance from the residence of each WIC participant to every California WIC-approved vendor was calculated. Geographic access was then summarized as the proximity of WIC-approved vendors (the distance to the nearest 1–5 vendors) and density (WIC-approved vendors/square mile) in buffers of 1, 2, 3, 4, and 5 miles around the residence of each survey respondent to capture potential differences at distances requiring different modes of transportation (e.g., walking, driving). To address potential confounding by Supplemental Nutrition Assistance Program (SNAP) benefit utilization, the same process was used to characterize access to SNAP-participating vendors in California.

### Covariates

Data available to characterize study participants include the child’s sex (male, female), and age (12–23 mo, 24–35 mo, 36–47 mo, 48–60 mo); the caregiver’s race, ethnicity, and language preference (categories of Asian, Black, Hispanic Spanish-speaking, Hispanic English-speaking, White, and other), educational attainment (completed less than high school, completed high school, completed more than high school) and employment status (currently working for pay: yes, no); and the number of all individuals (continuous), children aged <5 y (continuous), children aged 5 to 17 y (continuous), and WIC participants (continuous) in the household, household food security status assessed with the USDA 6-item Household Food Security Survey Module (insecure: low/very low food security, 2–6 affirmative responses; secure: high/marginal food security, 0–1 affirmative responses) [28], and SNAP participation (yes, no).

Contextual factors were also used to characterize the census tract of residence of survey respondents. The geographic density of all food vendors in the census tract of residence was used to characterize the total availability of food vendors regardless of WIC or SNAP approval status [29]. Population density and a neighborhood deprivation index for the census tract of residence were also calculated to characterize the neighborhood environment. The neighborhood deprivation index was calculated for census tracts, using data from the 2020 United States Census and 2015–2020 American Community Survey [30], [31], as the first component in a principal components analysis for the proportions of individuals with income <$100,000, with an income below the federal poverty line, on Temporary Assistance for Needy Families, on SNAP, on Medicaid, without a high school diploma (ages >25 y), without medical insurance, employed in a blue-collar profession, and currently employed; and proportions of those in a household that speaks a language other than English, that is female-headed, and that is owner-occupied [32]. Higher values for the neighborhood deprivation index indicate tracts with lower socioeconomic status.

### Statistical analysis

Respondent characteristics for the analytic sample were summarized with frequencies and proportions for categorical variables and with means and SDs for continuous variables. The distribution of WIC benefit redemption percentage in each benefit category was summarized with both the mean and SD and median and interquartile range (25th percentile, 75th percentile) for each food benefit category that was issued to the respondent’s household. The geographic density of WIC-approved and SNAP-approved vendors in 1 to 5 mile buffers around the residence (vendors/mile^2^) and the proximity of the closest 5 WIC-approved and SNAP-approved vendors from the residence (miles) were summarized with means and SDs.

The association between density of WIC-approved vendors (vendors/mile^2^ in 1–5 mile buffers around the residence) and WIC benefit redemption percentage was assessed in multivariable linear regression generalized estimating equation models, for overall redemption percentage and redemption percentage in each WIC food benefit category. Models accommodated clustering of respondents within local agency WIC programs and incorporated poststratification weights to adjust for survey nonresponse. Models were run separately for vendor density in buffers of 1 to 5 miles, and estimated the difference [95% confidence interval (CI)] in WIC benefit redemption percentage associated with 1 additional WIC-approved vendor/mile^2^ in the specific buffer. Each model included independent variables for child age category and sex; caregiver educational attainment, employment status, race/ethnicity, and language preference; household SNAP participation, household size, and the number of WIC participants in the household; mode of survey completion (phone or online); contextual factors for the census tract of residence including the geographic density of all food vendors, population density, and neighborhood deprivation index; and the geographic density of food vendors (vendors/mile^2^) that are WIC-approved and those that are SNAP-approved.

To estimate the difference (95% CI) in WIC benefit redemption percentage associated with greater proximity (i.e., with 1 fewer mile to the 1–5 closest WIC-approved vendors to the residence), a linear regression generalized estimating equation model was used, including independent variables for the distance to the 5 closest WIC-approved vendors in a single regression model, in addition to the distances to the 5 closest SNAP-approved vendors. A separate model was run for each WIC food benefit category, and models also included the same child, caregiver, household, and neighborhood covariates as the models for geographic density of vendors.

Respondents with missing data were excluded from the analysis [33]. Locations of respondent residences and access to vendors were determined in ArcGIS 10.8 (Environmental Systems Research Institute). Statistical analyses were conducted with SAS 9.4 (SAS Institute Inc.). *P* values of <0.05 were considered statistically significant.

## Results

The weighted analytic sample was primarily Hispanic (78.1%) (Table 1). Children were evenly distributed across sex (50.7%) and across age categories (12–23 mo: 24.8%; 24–35 mo: 28.5%; 36–47 mo: 26.8%; 48–60 mo: 19.9%). Most caregivers had completed high school or more education (80.1%), and less than half were working for pay (46.5%). Nearly half of the households were SNAP participants (44.0%), and household food insecurity was prevalent (39.6%). Households contained a mean of 4.5 individuals (SD: 1.6), with a mean of 1.4 WIC participants (SD: 0.6). The mean population density (100 people/mile^2^) of respondents’ neighborhoods was 101.5 (SD: 100.5), with a mean neighborhood deprivation index score of 1.9 (SD: 2.1). Comparison of the analytic sample with and without poststratification weights applied identified higher Spanish-language preference and lower respondent educational attainment in the unweighted sample.

**TABLE 1:** Characteristics of the analytic sample of respondents to the 2023 California Statewide WIC Survey (*n* weighted = 2173)

Variable	Weighted sample *N* = 2173	Unweighted sample *N =* 2201
Male, *n* (%)	1101 (50.7)	1111 (50.5)
Race/ethnicity-language, *n* (%)		
Non-Hispanic Asian	44 (2.0)	62 (2.8)
Non-Hispanic Black	109 (5.0)	108 (4.9)
Hispanic, English-speaking	1009 (46.4)	730 (33.2)
Hispanic, Spanish-speaking	689 (31.7)	997 (45.3)
Non-Hispanic other	91 (4.2)	212 (9.6)
Non-Hispanic White	230 (10.6)	92 (4.2)
Age category (mo), *n* (%)		
≤24	538 (24.8)	546 (24.8)
24–35	620 (28.5)	592 (26.9)
36–47	582 (26.8)	575 (26.1)
48–60	433 (19.9)	488 (22.2)
Caregiver educational attainment, *n* (%)		
<High school completion	434 (20.0)	560 (25.4)
Completed high school	830 (38.2)	832 (37.8)
>High school completion	910 (41.9)	809 (36.8)
Caregiver working for pay, *n* (%)	1011 (46.5)	977 (44.4)
Household SNAP participation, *n* (%)	956 (44.0)	953 (43.3)
Household food insecure, *n* (%)	860 (39.6)	885 (40.2)
Household composition, mean ± SD		
Total number of individuals	4.5 ± 1.6	4.6 ± 1.6
Children aged <5 y	1.3 ± 0.5	1.2 ± 0.5
Children aged 5–17 y	1.1 ± 1.2	1.1 ± 1.2
Number of WIC participants	1.4 ± 0.6	1.4 ± 0.6
Neighborhood characteristics,^1^ mean ± SD		
Population density (100 people/mile^2^)	101.5 ± 100.5	101.9 ± 100.4
Neighborhood deprivation index^2^	1.9 ± 2.1	1.9 ± 2.1

Abbreviations: SNAP, Supplemental Nutrition Assistance Program; TANF, Temporary Assistance for Needy Families; WIC, Special Supplemental Nutrition Program for Women, Infants, and Children.

1 Neighborhood of residence was assessed as the census tract in which the participant lived.

2 Neighborhood deprivation index was calculated at the census-tract level as the first component in a principal components analysis for the proportion of individuals with income <$100,000, with an income below the federal poverty line, on TANF, on SNAP, on Medicaid, without a high school diploma (ages >25 y), without medical insurance, employed in a blue-collar profession, currently employed; and that live in a household that speaks a language other than English, that is female-headed, and that is owner-occupied.

Households redeemed an average of 53.7% of all food benefits issued over the 12 mo before the survey (Table 2). Median redemption was highest for fruits and vegetables (75%), whole milk (75%), and reduced-fat milk (66.7%). Mean redemption was highest for whole milk (70.3%), fruits and vegetables (64%), and reduced-fat milk (62.5%). Yogurt, legumes, and whole grains were the lowest redeemed foods for both mean (41.4%, 46.7%, and 49.7%, respectively) and median redemption (33.3%, 35.4%, and 41.7%, respectively).

**TABLE 2:** Distribution of WIC benefit redemption in the 12 mo before the survey among respondents to the 2023 California Statewide WIC Survey (*n* = 2173)^1^

Food category	*n*	Mean ± SD	Median (IQR)
Overall	2173	53.7 ± 29.4	49.7 (26.9, 81.5)
Fruits/vegetables	2173	64.0 ± 33.7	75.0 (29.2, 100.0)
Whole milk	1077	70.3 ± 30.3	75.0 (50.0, 100.0)
Reduced-fat milk	1634	62.5 ± 33.9	66.7 (33.3, 100.0)
Eggs	2173	56.8 ± 33.7	58.3 (25.0, 91.7)
100% juice	2173	54.0 ± 33.6	50.0 (25.0, 90.0)
Cheese/tofu	2173	53.0 ± 34.1	50.0 (22.9, 88.9)
Breakfast cereal	2173	50.1 ± 32.8	41.7 (21.7, 83.3)
Whole grains	2173	49.7 ± 32.8	41.7 (22.9, 83.3)
Legumes	2173	46.7 ± 33.4	35.4 (16.7, 81.8)
Yogurt	2173	41.4 ± 32.8	33.3 (12.5, 66.7)

IQR = 25th percentile, 75th percentile.

Abbreviation: WIC, Special Supplemental Nutrition Program for Women, Infants, and Children.

1 WIC benefit redemption was calculated monthly as the percentage of issued food benefits that were redeemed (exchanged at a participating vendor for the specific eligible foods) and averaged across the 12 mo preceding the respondent-specific date of completion for the survey. Redemption ranges from 0% to 100%.

The mean WIC-approved vendor density was 0.9 vendors/mile^2^ (SD: 0.9) within a 1-mile radius of participants’ residences, decreasing to 0.5 vendors/mile^2^ (SD: 0.4) at the 5-mile buffer (Table 3). Mean SNAP-approved vendor densities were higher but followed a similar pattern, with 7.4 vendors/mile^2^ (SD: 6.9) within a 1-mile radius, decreasing to 4.3 vendors/mile^2^ (SD: 4.3) within a 5-mile radius. With respect to distance, the nearest WIC-approved vendor was located at a mean distance of 0.9 miles (SD: 1.5) from participants’ residences, increasing to 2.7 miles (SD: 4.8) to the 5th nearest vendor. SNAP-approved vendors were closer, with the nearest located 0.4 miles (SD: 0.6) and the 5th nearest at 0.8 miles (SD: 2.0).

**TABLE 3:** Measures of geographic access to WIC- and SNAP-approved vendors among respondents to the 2023 California Statewide WIC Survey (*n* = 2173).

Buffer radius	Mean vendor density^1^		Closest vendors	Mean vendor distance^2^	
	WIC-approved	SNAP-approved		WIC-approved	SNAP-approved
1 mile	0.91 ± 0.85	7.43 ± 6.78	1st closest	0.85 ± 1.53	0.36 ± 0.57
2 miles	0.70 ± 0.56	6.04 ± 5.37	2nd closest	1.41 ± 2.89	0.51 ± 1.01
3 miles	0.59 ± 0.47	5.22 ± 4.82	3rd closest	1.88 ± 3.77	0.63 ± 1.25
4 miles	0.52 ± 0.42	4.68 ± 4.49	4th closest	2.32 ± 4.57	0.74 ± 1.85
5 miles	0.47 ± 0.39	4.31 ± 4.25	5th closest	2.70 ± 4.79	0.82 ± 1.97

Abbreviations: SNAP, Supplemental Nutrition Assistance Program; WIC, Special Supplemental Nutrition Program for Women, Infants, and Children.

1 Vendor density around the residence was assessed as vendors/mile^2^ in circular buffers of 1–5 miles around the residence of study participants.

2 Distance to vendors was assessed as the geographic distance in miles from the residence to the 1st–5th nearest WIC- or SNAP-approved vendors.

Each additional vendor/mile^2^ in a 2-mile radius of participants’ residences was associated with 5.5% (95% CI: 0.6%, 10.4%) higher overall WIC benefit redemption (Table 4). The density of WIC-approved vendors within 1-, 3-, 4-, or 5-mile radius buffers was not significantly associated with overall WIC benefit redemption. The density of WIC-approved vendors within a 2-mile radius was significantly associated with redemption of select food categories: each additional vendor/mile^2^ was associated with 7.2% higher redemption of legumes (95% CI: 1.4%, 12.9%), 7.6% higher redemption of whole grains (95% CI: 1.7%, 13.5%), and 6.2% higher redemption of yogurt (95% CI: 0.7%, 11.7%). The density of WIC-approved vendors within a 1-mile radius and 4-mile radius was also significantly associated with higher redemption of yogurt [2.9% (95% CI: 0.2%, 5.6%) and 12.1% (95% CI: 0.5%, 23.8%), respectively]. No significant associations were observed for the density of WIC-approved vendors within any other buffer radius with redemption of any other food categories. The distance to the 3rd closest vendor was associated with 1.4% higher (95% CI: 0.4%, 2.5%) redemption of reduced-fat milk (Supplemental Table 1). No other significant associations between the distance to the nearest WIC-approved vendor and redemption of the overall benefits or any specific WIC food category were observed.

**TABLE 4:** Association between WIC vendor density (vendors/mile^2^) around the residence and WIC benefit redemption among respondents to the 2023 California Statewide WIC Survey (*n* weighted = 2173)^1^

WIC food category	Buffer radius				
	1 mile	2 miles	3 miles	4 miles	5 mile
Overall	1.7 (−0.6, 4.0)	5.5 (0.6, 10.4)	5.9 (−1.7, 13.4)	7.2 (−3.1, 17.6)	5.2 (−8.0, 18.4)
Fruits/vegetables	0.7 (−1.7, 3.2)	2.8 (−2.4, 7.9)	3.9 (−4.1, 11.8)	2.6 (−8.2, 13.5)	0.1 (−14.2, 14.3)
Whole milk	1.0 (−2.4, 4.3)	1.0 (−6.3, 8.3)	2.7 (−8.3, 13.7)	2.0 (−13.8, 17.8)	1.5 (−19.6, 22.6)
Reduced-fat milk	−0.3 (−3.3, 2.7)	4.1 (−2.3, 10.6)	4.8 (−5.1, 14.7)	7.6 (−6.0, 21.3)	7.9 (−9.8, 25.6)
Eggs	1.3 (−1.2, 3.9)	5.2 (−0.1, 10.5)	6.5 (−1.7, 14.7)	6.3 (−4.8, 17.3)	5.0 (−9.2, 19.2)
100% juice	1.9 (−0.8, 4.6)	5.0 (−0.7, 10.7)	3.3 (−5.6, 12.2)	5.2 (−7.1, 17.5)	3.2 (−12.5, 18.8)
Cheese/tofu	2.0 (−0.8, 4.7)	5.1 (−0.7, 10.9)	6.4 (−2.4, 15.2)	7.6 (−4.3, 19.4)	5.5 (−9.6, 20.6)
Breakfast cereal	2.4 (−0.3, 5.0)	4.4 (−1.2, 10.0)	4.0 (−4.6, 12.6)	4.2 (−7.5, 16.0)	−1.1 (−16.4, 14.1)
Legumes	2.0 (−0.8, 4.7)	7.2 (1.4, 12.9)	8.1 (−0.5, 16.7)	10.4 (−1.9, 22.6)	6.7 (−9.1, 22.4)
Whole grains	2.1 (−0.7, 4.9)	7.6 (1.7, 13.5)	6.7 (−2.5, 15.8)	9.7 (−2.9, 22.3)	6.7 (−9.0, 22.4)
Yogurt	2.9 (0.2, 5.6)	6.2 (0.7, 11.7)	7.2 (−1.4, 15.8)	12.1 (0.5, 23.8)	12.4 (−2.8, 27.7)

Abbreviations: SNAP, Supplemental Nutrition Assistance Program; WIC, Special Supplemental Nutrition Program for Women, Infants, and Children.

1 WIC benefit redemption reflects the percentage of WIC foods, in specific benefit categories or across all categories, issued to the household of the survey respondent that were exchanged for the issued foods at WIC-participating vendors over the 12 mo before the completion of the survey. The association of geographic density of WIC-approved vendors in variably sized buffers with WIC benefit redemption was estimated in generalized estimating equation linear regression models that incorporated statistical weights to correct for survey nonresponse, accommodated clustering of individuals within local agency WIC program, and included independent terms for child age category and sex; caregiver (adult who provides care for WIC-participating child, with 99% being a biological parent) educational attainment, employment status, and race/ethnicity and language preference; household SNAP participation, household size, and the number of WIC participants in the household; mode of survey completion; contextual factors for the census tract of residence including the geographic density of all food vendors, population density, and a neighborhood deprivation index; and the geographic density of food vendors (vendors/mile^2^) that are WIC-approved and those that are SNAP-approved. Associations between 1-additional WIC-approved vendor/mile^2^ in specified buffers around the residences of the study participant with WIC benefit redemption are presented as the difference (95% confidence interval) in WIC benefit redemption percentage.

## Discussion

Greater geographic density of vendors in a 2-mile buffer around the residence was associated with higher overall benefit redemption and higher redemption of legumes, whole grains, and yogurt. These findings are in alignment with results for the SNAP program, which reported that the vendor landscape was associated with both diet quality and benefit redemption [34], [35], [36]. Specifically, SNAP participants in states with lower geographic density of approved vendors redeemed their benefits more slowly throughout the month of issuance [36], and households with lower geographic access to SNAP-approved vendors had lower diet quality assessed with the Healthy Eating Index [34]. Prior findings of associations between the density of vendors around the residence and weight status among WIC participants in Los Angeles County, California, also align with the present results. In the first report, lower weight-for-length z-scores (i.e., healthier weight status) were found among WIC-participating children living in households with a geographic density of health food outlets around 0.7 vendors/mile squared [20]. These healthy food outlets include grocery stores, produce vendors, and supermarkets (i.e., the types of vendors most likely to be WIC-approved) and suggest that insufficient access to healthy food outlets may contribute to less healthy dietary practices and greater adiposity among WIC participants [20]. In the second report, the association between the 2009 WIC food package revision and lower obesity risk was stronger for children in neighborhoods with higher healthy food outlet density [22], which could be explained by greater access to WIC-approved vendors in neighborhoods with higher healthy food outlet density leading to higher WIC benefit utilization. Unhealthy outlet density significantly modified the association among female children [22].

Distance to each of the nearest 5 WIC-approved vendors around the residence was not associated with redemption of overall WIC foods, nor with redemption of specific food benefit categories issued to participating children ages 1 to 4 y. This finding aligns with prior research in low-income urban communities that identified that preferred vendors for household food purchasing are often not the nearest to the residence [37], and that other factors, including food prices, may be more important to shoppers than proximity to the residence [38]. The findings of the present study are in contrast to those of a prior study of SNAP-participating households, which found higher diet quality among SNAP participants who lived closer to SNAP-approved vendors and concluded that this is because those living closer to SNAP-approved vendors (regardless of type, including grocery stores, supermarkets, superstores, and convenience stores) had a greater opportunity to redeem their benefits to support healthy diets [34]. The contrasting results may be attributable to differences in the operationalization of proximity (as the continuous distance in miles to the nearest WIC-approved vendors in the present study as opposed to a categorical indicator for distance to the nearest SNAP-approved vendor used in the prior study) [34], to differences in study outcomes (redemption and diet quality), or to differences in the 2 programs and the types of individuals who participate in each [39], [40].

The absence of associations between density of WIC-approved vendors with overall or category-specific WIC benefit redemption (except for yogurt redemption) in buffers of 1, 3, 4, and 5 miles was unexpected. In prior research using the Reasons for Geographic Differences in Stroke cohort on the relationship between access to food vendors and diet quality (dietary inflammation score), consistent associations were found between vendor access in buffers with radii of 1 to 10 miles around the residence [41]. The absence of an association between vendor density and benefit redemption in the 1, 3, 4, and 5-mile buffer radii in the present study may reflect that the appropriate geographic context is the 2-mile radius [42], [43], or alternatively that there was insufficient variability in WIC vendor density in the smallest or largest geographic units to identify associations with benefit redemption.

Prior research on food purchasing behaviors has found that households often do not use the most proximate food vendor for purchasing food [44]. Rather, the quality of the nutrition environment in stores is associated with how far households travel to do their food purchasing [37]. Other studies have reported that prices for healthy foods outweigh the proximity of vendors in the decision for where a household will do its food purchasing [38]. Together, these prior findings suggest that having a greater density of WIC-approved vendors around the residence may be associated with higher redemption because households are more likely to find a vendor that meets their preferences within an appropriate distance to travel to redeem their WIC benefits. The lack of association between distance from the residence to the nearest 5 WIC-approved vendors may also be a function of other factors, such as food prices taking priority over proximity when selecting among vendors [37], [38]. WIC food benefits, with the exception of fruits and vegetables, are issued as quantity amounts (e.g., 1 dozen eggs), which means that participants are not exposed to the price of redeeming their WIC foods at a participating vendor. However, given that many WIC participants report doing other food purchasing at the WIC vendor of choice, it is possible that the perceived value of foods purchased with other resources at the same vendor may play a role in the choice of vendor.

Respondents in this study redeemed an average of 54% of their WIC food benefits across the 12 mo before the survey, which is lower than in some recent studies using only WIC administrative data [9], [45]. Overall redemption may be lower in this sample because of the 2022 infant formula supply crisis during the 12 mo preceding the study [46], [47] and the large increase in the WIC cash value benefit [48], which may have led to lower percentage redemption for fruits and vegetables and infant formula and contributed to lower average food benefit redemption. This study was also conducted before the introduction of the new WIC food package in April 2026, which reduced issuance amounts for some lower-redemption food categories that were issued in excess of Dietary Guidelines for Americans intake recommendations [49], [50]. Future studies should consider evaluating WIC redemption based on geographic proximity and density longitudinally to see whether changes occurred after the new WIC food package was introduced.

This study has a number of noteworthy strengths, including a large random sample of study-eligible WIC-participating households with statistical weighting incorporated to mitigate nonresponse. Geographic locations of participant residences and WIC-approved vendors allowed the assessment of density and proximity of vendors for each household, with the ability to explore multiple geographic units to accommodate potentially different associations at walking (<1 mile) and driving (1–<5 miles) distances around the residence. Redemption data were available for the 12 mo before the survey, allowing for characterization of long-term redemption behaviors, and a robust set of covariates was available to adjust for potential confounding factors. Study limitations include the observational and cross-sectional design, preventing causal inference and determination of directionality of identified associations. The uncertain geographic context problem [51] may also impact the findings, particularly if the vendor context around the residence (as opposed to around the place of work, or along the route from the residence to work) is not the relevant exposure for WIC benefit redemption. This study was conducted among WIC-participating households in California with a study-eligible child aged 1 to 4 y, and caution should be used when extrapolating results to demographically distinct populations or those with differing WIC vendor contexts.

Higher density of WIC-approved vendors around the residence is associated with higher redemption of WIC food benefits overall, and for specific food benefit categories that have lower average redemption (legumes, whole grains, and yogurt). These findings from a representative sample of WIC-participating households with a participating child aged 1 to 4 y in California suggest that vendor access may contribute to differences in benefit redemption observed by participant and household characteristics [9], [45]. Vendor access may therefore also contribute to disparities in participant outcomes that are associated with benefit redemption, including continued program participation [10], [13] and child diet [12]. Further research is needed to establish whether geographic access to WIC-approved vendors is associated with dietary intake and food security among participating households. Quasi-experimental research approaches leveraging changes in the contents and amounts of issued WIC food benefits could provide causal evidence for associations between vendor access and benefit redemption. If lower vendor access is associated with lower food security and diet quality, policies to ameliorate the impacts of low geographic access, such as online ordering, grocery delivery, or increasing vendor counts in low-access areas, should be considered to best support the nutrition and health of WIC participants.

## Author contributions

The authors’ responsibilities were as follows – CEA: conducted the research and the statistical analysis and took primary responsibility for the final content; CEA, HC, AMC: drafted the manuscript; SEW, LEA, HC, AMC: provided critical revision of the manuscript for important intellectual content; CEA, SEW: conceptualized and designed the study; all authors: read and approved the final version and took responsibility for the accuracy and integrity of the results.

## Data availability

Data described in the manuscript include confidential administrative data and will not be made publicly available due to an agreement with the California Department of Public Health WIC Division.

## Declaration of generative AI and AI-assisted technologies in the writing process

The authors declare that no generative AI or AI-assisted technologies were used in the writing of this manuscript.

## Funding

This study was supported by a seed grant (2025-67017-45034) from the Agriculture and Food Research Initiative (AFRI) of the National Institute of Food and Agriculture (NIFA) and USDA/NIFA Hatch funding (project # CA-D-NTR-2946-H). The funder had no role in the design or conduct of the study, the analysis of the data, the drafting of the manuscript, or the decision to submit for publication.

## Conflict of interest

The authors report no conflicts of interest.
