# Supplementary material for: Associations of Geographic Access to Special Supplemental Nutrition Program for Women, Infants, and Children–Approved Vendors with Special Supplemental Nutrition Program for Women, Infants, and Children Benefit Redemption in California
Source: Curr Dev Nutr. 2026 Oct 23;10(11):109558. doi: 10.1016/j.cdnut.2026.109558 (PMC13763424; doi:10.1016/j.cdnut.2026.109558)
Supplement: Supplementary file 1 [file mmc1.docx]

**Supplemental Table 1**. Percent difference in WIC benefit redemption percent in the 12 months before the 2023 California Statewide WIC Survey associated with a 1-mile shorter distance to the X^th^ WIC vendor (n weighted=2,173).^a^

| WIC food category | Vendor proximity rank | | | | |
| --- | --- | --- | --- | --- | --- |
|  | 1^st^ closest | 2^nd^ closest | 3^rd^ closest | 4^th^ closest | 5^th^ closest |
| Overall | 0.7 (-0.7, 2.1) | 0.1 (-0.7, 0.8) | 0.2 (-0.6, 1.1) | -0.6 (-1.9, 0.7) | 0.5 (-0.7, 1.8) |
| Fruits/vegetables | 1.3 (-0.3, 2.9) | 0.1 (-1.2, 1.5) | 0.3 (-0.8, 1.3) | -0.3 (-1.4, 0.9) | -0.1 (-1.2, 1.0) |
| Whole milk | 0.5 (-1.4, 2.4) | -0.2 (-2.3, 1.9) | 0.7 (-1.2, 2.5) | -1.6 (-3.7, 0.5) | 0.6 (-1.0, 2.2) |
| Reduced fat milk | -0.4 (-2.7, 2.0) | 0.5 (-0.7, 1.6) | **1.4 (0.4, 2.5)** | 0.1 (-2.2, 2.3) | -0.5 (-2.5, 1.4) |
| Eggs | 0.9 (-0.8, 2.6) | 0.3 (-0.7, 1.4) | -0.5 (-1.8, 0.8) | -0.6 (-2.1, 0.9) | 0.8 (-0.7, 2.2) |
| 100% juice | 0.3 (-1.3, 1.9) | 0.2 (-0.7, 1.1) | 0.0 (-1.2, 1.2) | -0.6 (-2.0, 0.9) | 0.6 (-0.7, 1.9) |
| Cheese/tofu | 0.3 (-1.5, 2.1) | 0.3 (-0.6, 1.2) | -0.0 (-1.1, 1.1) | -0.5 (-2.1, 1.1) | 0.5 (-1.0, 2.0) |
| Breakfast cereal | 0.3 (-1.4, 2.0) | -0.0 (-0.9, 0.9) | 0.5 (-0.5, 1.5) | -0.7 (-2.6, 1.3) | 0.3 (-1.5, 2.1) |
| Legumes | 0.4 (-1.2, 1.9) | -0.0 (-0.9, 0.8) | 0.3 (-1.0, 1.5) | -0.9 (-2.3, 0.6) | 0.8 (-0.6, 2.1) |
| Whole grains | 1.0 (-0.5, 2.5) | -0.0 (-1.0, 0.9) | -0.3 (-1.8, 1.2) | 0.0 (-1.6, 1.6) | 0.6 (-0.8, 1.9) |
| Yogurt | 0.0 (-1.8, 1.8) | 0.4 (-0.5, 1.4) | -0.3 (-1.8, 1.1) | -0.5 (-2.2, 1.2) | 1.0 (-0.5, 2.4) |

^a^ WIC benefit redemption reflects the percent of WIC foods, in specific benefit categories or across all categories, issued to the household of the survey respondent that were exchanged for the issued foods at WIC participating vendors over the 12 months before the completion of the survey. The association of distance to the 1^st^ to 5^th^ nearest WIC vendors with WIC benefit redemption was estimated in generalized estimating equation linear regression models which incorporated statistical weights to correct for survey non-response, accommodated clustering of individuals within local agency WIC program, and included independent terms for child age category and gender; caregiver (adult who provides care for WIC-participating child, with 99% being a biological parent) educational attainment, employment status and race/ethnicity and language preference; household SNAP participation, household size, and the number of WIC participants in the household; mode of survey completion; contextual factors for the census tract of residence including the geographic density of all food vendors, population density, and a neighborhood deprivation index; and the distances to the 1^st^ to 5^th^ nearest WIC-approved vendors and the 1^st^ to 5^th^ nearest SNAP-approved vendors. Associations between 1-less mile from the residence of the study participant to the nearest X^th^ WIC-approved vendor with WIC benefit redemption are presented as the difference (95% confidence interval) in WIC benefit redemption percent.
